# Influence of the *ACTN3* R577X genotype on the injury epidemiology of marathon runners

**DOI:** 10.1371/journal.pone.0227548

**Published:** 2020-01-28

**Authors:** Victor Moreno, Francisco Areces, Diana Ruiz-Vicente, José M. Ordovás, Juan Del Coso

**Affiliations:** 1 Sports Research Centre, Miguel Hernandez University of Elche, Alicante, Spain; 2 Exercise Physiology Laboratory, Camilo José Cela University, Madrid, Spain; 3 USDA ARS, Human Nutrition Research Center on Aging at Tufts University, Boston, MA, United States of America; 4 IMDEA Food Institute, CEI UAM + CSIC, Madrid, Spain; 5 Centre for Sport Studies, Rey Juan Carlos University, Fuenlabrada, Madrid, Spain; Universidade Federal de Mato Grosso do Sul, BRAZIL

## Abstract

A common single nucleotide polymorphism in the *ACTN3* gene might result in the complete deficiency of α-actinin-3 (i.e., XX genotype). It has been found that *ACTN3* XX individuals have several traits related to lessened muscle performance. This study aimed to determine the influence, if any, of *ACTN3* genotypes on injury incidence of marathoners during the year preceding to participating in a competitive marathon race. Using a cross-sectional experimental design, the type and conditions of sports injuries were documented for one year in a group of 139 marathoners. Injuries were recorded following a consensus statement on injuries in Athletics. Afterward, *ACTN3* genotyping was performed, and injury epidemiology was compared among RR, RX, and XX genotypes. The distribution of the RR/RX/XX genotypes was 28.8/42.8/23.5%, respectively. A total of 67 injuries were recorded. The frequency of marathoners that reported any injury during the previous year was not different across the genotypes (55.0/38.8/40.6%, *P* = 0.241). Although the overall injury incidence was not different among genotypes (2.78/1.65/1.94 injuries/1000 h of running, *P* = 0.084), the likelihood of suffering an injury was higher in RR than in RX (OR = 1.93: 95%CI = 0.87–4.30), and higher than in XX (OR = 1.79: 0.70–4.58). There was no difference in the conditions, severity, body location, time of year, or leading cause of injury among genotypes. However, XX presented a higher frequency of sudden-onset injuries (*P* = 0.024), and the OR for muscle-type injuries was 2.0 (0.51–7.79) times higher compared to RR runners. Although XX marathoners did not have a higher overall incidence of injury, the OR in these runners for muscle-type injuries was superior to RR and RX runners. The likelihood of suffering a muscle injury, especially with a sudden-onset, was twice in XX than in RR endurance runners.

## Introduction

Endurance running is one of the most widespread sports around the world [1]. Due to its health benefits, low cost and ease of implementation, endurance running has gained popularity among sports practitioners in recent years, especially in those seeking to complete a marathon race. Consequently, the number of amateur endurance runners participating in marathon races has rocketed over the past decade [2].

Running a marathon is a very challenging physical activity that involves both concentric and eccentric muscle actions repeated for 2 to 6 hours, depending on the level of the runner [3]. Thus, the training for a marathon can lead to frequent lower limb injuries in the months before the competition [4]. In those preparing for a marathon, more than half suffered an injury in the year before the race, and the calf, knee, and thigh are the most recurrent body locations for such injuries [5]. Although running-related injuries are affected by a myriad of factors and they can be multifactorial, athlete’s age, training volume, history of previous injuries and running kinematics have been considered as the main determinants for the likelihood of suffering endurance running injuries [6, 7]. Yet, the influence of genetics on the rate and type of muscle injuries developed by endurance runners has not been investigated.

A common genetic single nucleotide polymorphism (SNP)—*ACTN3* R577X (rs1815739)—influences muscle performance [8] and might affect injury incidence in endurance runners. This SNP leads to the replacement of an arginine (R) base with a premature stop codon (X) that halts the production of functional α-actinin-3 in fast-type muscle fibers. Thus, individuals with the *ACTN3* XX genotype have a deficiency of α-actinin-3, an actin-binding protein with a pivotal role in muscle structure and metabolism [9]. In contrast, RX and RR individuals can express α-actinin-3 in fast-type muscle fibers, although recent evidence suggests dose-dependency for α-actinin-3 expression in RX *vs*. RR individuals [10]. The deficiency of α-actinin-3 has no clinical impact, but it has been demonstrated that it negatively affects those involved in sprint-based and power-based sports [11], likely due to decreased capacity of muscle fiber to produce force in the absence of α-actinin-3 [12]. This finding is based on several studies that showed a higher frequency of the *ACTN3* RR genotype in elite sprint/power athletes when compared with control groups composed of non-athletes (see reference [9] for a more concise analysis). On the contrary, a higher representation of the *ACTN3* XX genotype has been found in some populations of elite endurance athletes [13, 14], but this finding has not been replicated in other cohorts of endurance athletes [9]. Thus, current evidence suggests that XX genotype might negatively influence elite sprint/power-based exercise with little or no effect on endurance-based exercise.

A recent investigation with amateur marathon runners found that XX individuals produce less muscle force, have lower thigh fat-free mass volume and have higher percentages of body fat [15] than RR counterparts. In contrast, runners with the XX genotype had higher muscle flexibility and ankle dorsiflexion than runners with the RR or RX genotype [15]. These data suggest that α-actinin-3 deficiency, due to XX homozygosity in the *ACTN3* R577X polymorphism, might induce both positive and negative phenotypes for endurance running contributing to an explanation for why a loss-of-function genetic variant has been positively selected through evolution. Nevertheless, the association of these exercise phenotypes with injury epidemiology has not been previously investigated. Interestingly, XX runners are more prone to develop high levels of exercise-induced muscle damage in a marathon race [16] or in a half-ironman race [17], which suggests that XX genotype might be associated to a higher prevalence muscle-type injuries during endurance running. Although there is growing evidence to suggest that the *ACTN3* R577X polymorphism might affect the incidence of injury in sports [18], the evidence is still inconsistent.

In soccer, players with the XX genotype were more prone to suffer non-contact musculoskeletal soft-tissue injuries than players with the RR or the RX genotype [19, 20], although this association has not been found in runners. In contrast, other investigations have found that R-allele athletes had an increased likelihood of suffering non-contact muscle injuries during different sports-activities [21] and they have increased passive lower leg stiffness compared to XX counterparts [22] that might predispose to overuse injuries, especially in amateur endurance runners [23]. The most consistent finding has been the positive association between the XX genotype and ankle injuries [24–26] that suggests a negative impact of the XX genotype on joint and ligament injuries.

To our knowledge, there is no information about the effect of *ACTN3* genotypes on the incidence and type of non-contact injuries in endurance runners. For this reason, the present study aimed to determine the influence of *ACTN3* genotypes on injury incidence of marathoners during the year preceding to participating in a competitive marathon race. Our central hypothesis was that, compared with their RR counterparts, *ACTN3* XX runners would have a higher overall incidence of injuries.

## Methods

### Participants

One hundred and thirty-nine healthy experienced amateur marathon runners volunteered to participate in this study. Participants were recruited by email from a pool of runners that had participated in previous investigations. Age, main morphological characteristics and training routines of the participants in this investigation are shown in Table 1.

**Table 1:** Age, anthropometric characteristics, running experience and training status of marathoners with different *ACTN3* R577X genotypes. Data are mean ± SD.

*Variable (units)*	RR	RX	XX	*P* value
n (frequency)	40 (28.8%)	67 (48.2%)	32 (23.0%)	-
Men/women (frequency)	34/6 (85.0/15.0%)	58/9 (86.6/13.4%)	27/5 (84.4/15.6%)	0.950
Age (years)	41.3 ± 10.2	40.3 ± 8.8	40.7 ± 9.8	0.874
Body mass (kg)	69.6 ± 8.3	71.6 ± 10.8	72.8 ± 10.5	0.407
Body height (m)	1.72 ± 0.07	1.73 ± 0.08	1.72 ± 0.10	0.658
Body mass index (kg/m^2^)	23.6 ± 1.8	24.0 ± 1.9	24.5 ± 1.5	0.269
Experience (years)	8.7 ± 7.3	8.2 ± 7.8	8.3 ± 6.0	0.946
Training volume (km/wk)	50.6 ± 14.0	52.6 ± 17.0	51.7 ± 16.9	0.852
Training sessions (n/wk)	4.1 ± 1.0	4.0± 0.9	3.9 ± 1.1	0.861
Training volume (h/year)	246 ± 69	250 ± 77	223 ± 53	0.199
2018 marathon race time (min)	237 ± 37	236 ± 44	244 ± 27	0.499

### Ethical statement

Before enrolment, each participant was informed about the risks and discomforts associated with the investigation and signed an informed consent document. The study was approved by the Camilo Jose Cela University Ethics Committee following the latest version of the Declaration of Helsinki. Participants' rights and confidentiality were protected during the whole experiment, and the genetic information was used only for the purposes included in this investigation.

### Experimental design

This investigation is a cross-sectional study to determine the effect of the *ACTN3* R577X genotype (RR vs. RX vs. XX) on the incidence of lower limbs sport-related injuries suffered by amateur marathon runners during the previous 12 months. For this investigation, enrolled participants completed an *ad hoc* questionnaire about the non-contact injuries sustained during the year before competing in a marathon race, following the design proposed by Van Middelkoop et al. [5] Only injuries resulting from their training routines or competitions in endurance running activities were recorded. The questionnaire was based on the consensus statement on injury definitions and data collection in epidemiological studies in athletics [27]. The effect of the *ACTN3* R577X genotype in several exercise phenotypes such as anthropometry, force production, muscle flexibility and energy cost of running has been recently published using a subset of the individuals included in this investigation [15].

### Injury questionnaire

A recordable injury was defined as a physical complaint or visible damage to any part of the lower limbs sustained by an athlete and assessed by a qualified medical/healthcare practitioner [27]. Injuries were recorded only if they impeded the athlete in taking part in endurance running training or competition the day after the incident (i.e., time-loss injuries). All traumatic injuries, defined as a condition caused by an identifiable single external transfer the energy, such as the ones caused by a fall or due to contact with an obstacle or another athlete, were discarded. This is because the cause of the traumatic injuries has not been previously related to the *ACTN3* R577X genotype. The questionnaire included:

#### Baseline information

Athletes’ age, gender, height, weight, training volume (hours, sessions, and distance per week) and endurance running experience were recorded at the beginning of the questionnaire. With this data, the number of hours devoted to endurance running for the whole year was calculated.

#### Injury conditions

A clear definition of the injury, as expressed above, was specified, and it was requested whether the injury was sustained during a training routine or competition. The injury rate per 1000 hours of endurance running was calculated by numbers of injuries divided by the number of training hours in the year [27]. The onset of each injury was classified as sudden onset when the disability developed during minutes or seconds, or as a gradual onset injury, when the disability developed during hours, days, or more.

#### Injury severity

The assessment of severity started on the following day to the disability till full recovery determined by expert medical opinion, and it was classified as minor (1–7 days), moderate (8–28 days), or severe (>28 days) [27].

#### Body location and injury type

The lower-limb location of the injury was classified as hip/groin, thigh, knee, lower leg, ankle, foot/toe, and Achilles tendon. The type of injury was classified as strain/muscle injuries, tendinosis/tendinopathies, bone injuries, sprain/ligament injuries, and nerve injuries. Concussions, traumatic fractures, dislocation/subluxations, contusions, skin lesions, and dental injuries were not included as injury type as they were related to traumatic injuries.

#### Season

The time of the injury was classified by the season as pre-season when the injury occurred in the period immediately before the start of a new season, or in-season when it occurred during the training period for a specific competition. Also, the time of injury was classified by summer, autumn, winter, and spring.

#### Possible cause

To determine the possible cause for the development of the injury, the questionnaire included a section to indicate if the injury coincided with an abrupt increase in training volume or intensity (i.e., excessive load), a change of training surface, a change of running shoes, aspects related to biomechanics (e.g., new orthotic insoles) or if the cause was unknown.

### Genetic testing

At the time of the recollection of injury data, buccal swabs were collected and DNA was isolated using an organic-based DNA extraction method adapted to Amicon® Ultra 0.5-mL columns, including a final concentration step to 50 μL [28]. To avoid contamination, recommendations for molecular genetics laboratories were followed, including physically-isolated work area laboratories for each process (sample manipulation and extraction). In addition, reference samples (internal controls, blank samples, and negative controls) and contamination monitoring in all steps were included. Positive controls for all genotypes were obtained from the Mexican branch of the CANDELA Consortium. Genotyping of *ACTN3* rs1815739 polymorphism (c.1858C>T; p.R577X) was conducted using a TaqMan SNP Genotyping Assay (Assay ID: C___590093_1_; Applied Biosystems, Foster City, CA, USA) and the reaction was performed in an Applied Biosystems 7500 Fast Real-Time PCR System (Applied Biosystems). The results were analyzed using 7500 Software v2.0.5 (Applied Biosystems).

### Statistical analysis

Data on injury incidence were transferred from the questionnaire to an *ad hoc* database designed for this research. Data was transferred by one author (VM), and then they were checked for accuracy by another author (JDC). The normality of each variable was initially tested with the Kolmogorov-Smirnov tests, and parametric/non-parametric statistics were performed for normally/non-normally distributed variables, respectively. For the continuous variables, group comparisons (RR vs. RX vs. XX) were performed using a one-way analysis of variance (ANOVA) or Kruskal-Wallis tests. For the variables presented as frequency, the differences in distribution were identified with crosstabs and Chi-Square tests, including adjusted standardized residuals. Briefly, it was considered that a genotype had a distribution in the conditions of injury different from expected when its distribution was > or < the critical value of Z (i.e., 1.96). Finally, the odds ratio (OR) and 95% confidence interval (CI) of suffering an injury was calculated for the following pairwise comparisons: RR-RX, RR-XX, and RX-XX. The data were analyzed with the statistical package SPSS version 20.0 (SPSS Inc., Chicago, IL, USA). The significance level was set at *P* < 0.05.

## Results

From the study participants, 61 athletes (44.2%) reported at least one injury during the year before the marathon race (total 67 injuries, with a range of 1 to 3 injuries per year). The overall injury incidence was 1.98 injuries per 1000 h of endurance running. Table 2 contains information about the injuries detail in the study sample. Briefly, 83.6% of the injuries were sustained during training, 52.5% were classified as overuse injuries, and 52.5% required more than 28 days for a full recovery. From the total, 22.6% were strain/muscle injuries, 36.1% tendinosis/tendinopathies, 9.8% bone injuries, 21.3% sprain/ligament injuries, and 6.6% nerve injuries. The foot (23.0%) and the lower leg (21.3%) were the most common location for the injury. From the total, 88.5% of the injuries were sustained during the in-season period to prepare the marathon race, and 41.0% occurred during winter. Finally, an excessive load was the most common cause of the injury. All the reported injuries required medical attention by a qualified medical/healthcare practitioner, and they were diagnosed by a physician (39.3% of the injuries), a physiotherapist (49.2%), or other medical professionals (11.5%). From the total, 21.3% of the injures were confirmed by echography, 18.0% by magnetic resonance imaging, 11.5% by X-rays and the remaining 49.2% were diagnosed during the clinical visit with no complementary testing.

**Table 2:** Conditions of injury for *ACTN3* RR, RX and XX marathon runners. Data are frequencies in percentage for each group.

		All	RR	RX	XX	*P* value
Injury conditions						
	Training	83.6	77.3	88.5	84.6	0.642
	Competition	16.4	22.7	11.5	15.4	
Mode of onset						
	Sudden	47.5	45.5	34.6	76.9*	0.024
	Gradual	52.5	54.5	65.4	23.1*	
Injury severity						
	Minor	21.3	22.7	19.2	23.0	0.410
	Moderate	26.2	31.8	15.4	38.5	
	Serious	52.5	45.5	65.4	38.5	
Body location						
	Hip/Groin	11.5	9.1	7.7	23.1	0.860
	Thigh	9.8	0.0	19.2	7.7	
	Knee	14.8	18.2	11.5	15.4	
	Lower leg	21.3	13.6	23.1	30.8	
	Ankle	14.8	18.2	11.5	15.4	
	Foot/toe	23.0	31.8	23.1	7.7	
	Achilles tendon	4.9	9.1	3.8	0.0	
Season						
	Pre-season	11.5	4.5	19.2	7.7	0.430
	In-season	88.5	95.5	80.8	92.3	
Time of year						
	Summer	8.2	4.5	15.4	0.0	0.864
	Autumn	23.0	18.2	23.1	30.8	
	Winter	41.0	40.9	38.5	46.2	
	Spring	27.9	36.4	23.1	23.1	
Possible cause						
	Excessive load	59.0	54.5	53.8	76.9	0.434
	Surface	13.1	4.5	26.9	0.0	
	Shoe	11.5	18.2	7.7	7.7	
	Biomechanics	1.6	0.0	3.8	0.0	
	Unknown	14.8	22.7	7.7	15.4	

(*) Different from expected at *P* < 0.05.

**Fig 1:**
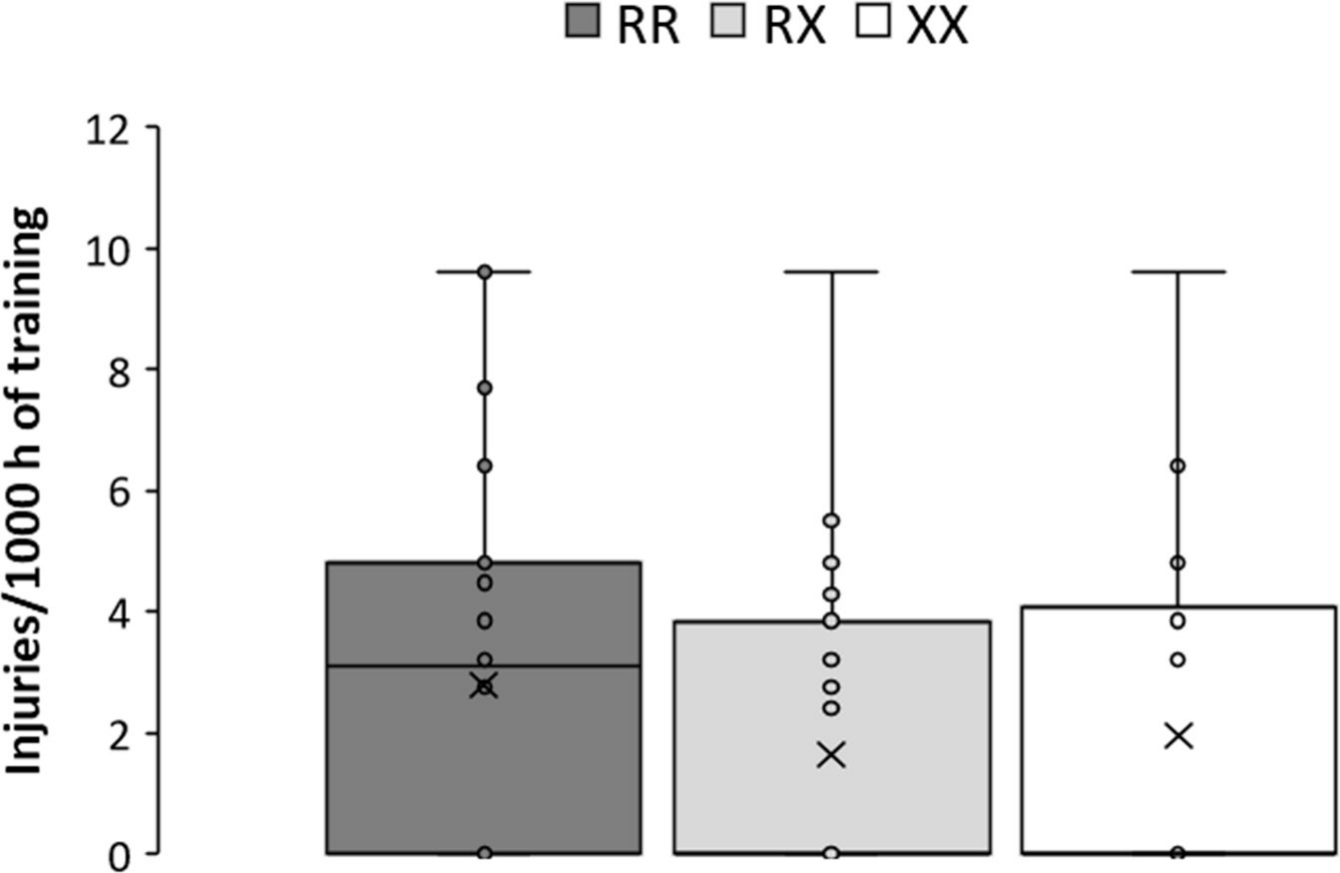
Box-and-whisker plots for injury rates in *ACTN3* RR, RX and XX amateur marathon runners. The cross depicts the mean value while the lower, middle and upper lines of the box represent the 25%, 50% and 75% percentile. Whiskers represent 1.5 × interquartile range.

The frequency of athletes that reported an injury during the previous year was not different across the genotypes (55.0/38.8/40.6% for RR/RX/XX genotypes, respectively, *P* = 0.241). However, the OR of suffering any injury was 1.93 (95%CI = 0.87–4.30) for the RR-RX comparison, 1.79 (0.70–4.58) for the RR-XX comparison and 0.93 (0.39–2.19) for the RX-XX comparison. RR athletes had an OR of 1.88 (0.90–3.95) for the likelihood of suffering any injury in contrast to X-allele carries. Fig 1 depicts medians and percentiles for the injury rate in RR, RX, and XX runners. Overall, there was no difference between genotypes in the injury rate (mean = 2.78/1.65/1.94, *P* = 0.084; median = 3.1/0.0/0.0 injuries per 1000 h of endurance running for RR/RX/XX, respectively, *P* = 0.128) but the injury rate was higher in RR vs X-allele carriers (*P* = 0.049).

**Fig 2:**
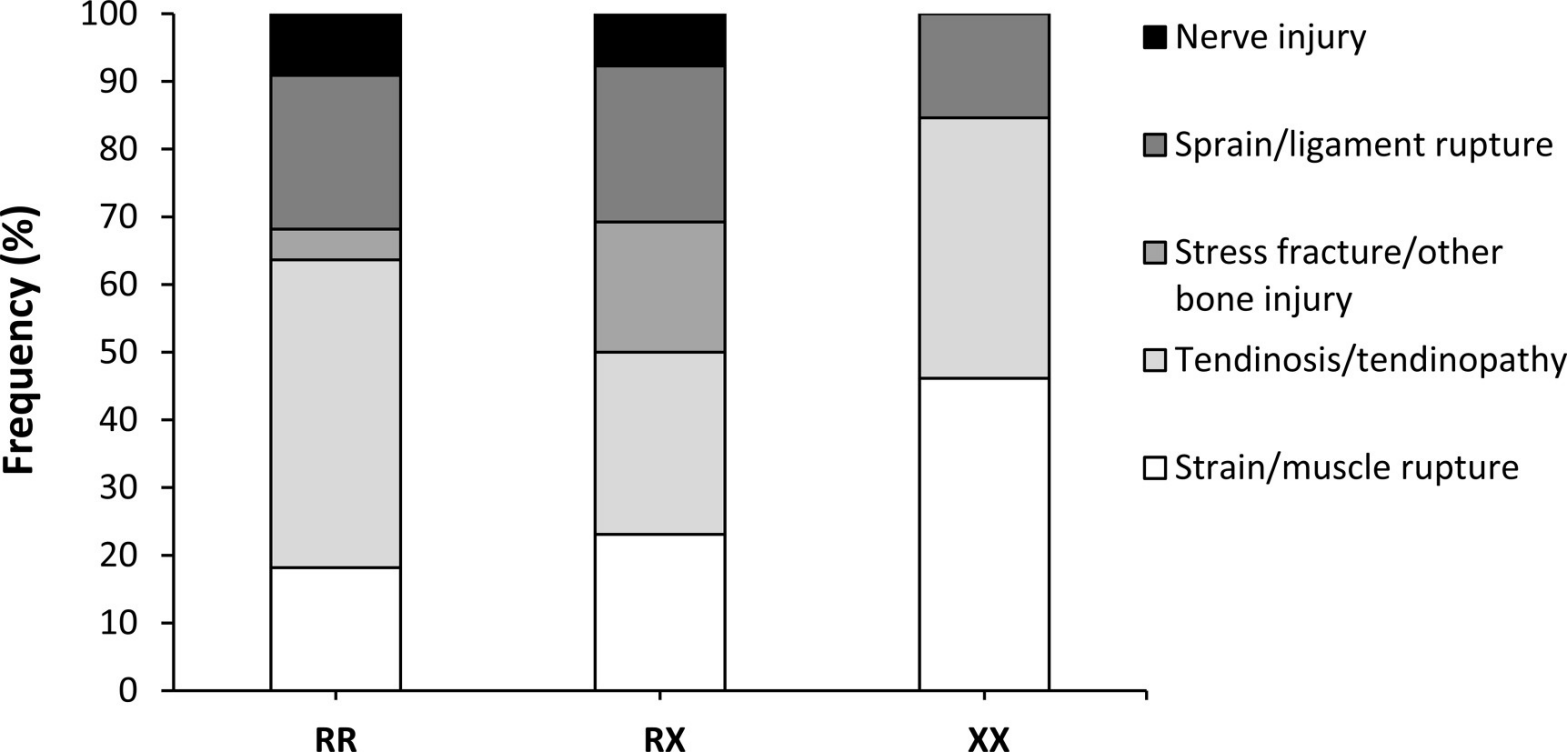
Type of injury in *ACTN3* RR, RX and XX amateur marathon runners. Data are the frequency for each type of injury from the total of injuries in each genotype.

There was no difference in the conditions, severity, body location, season, time of year, or cause of injury among genotypes (Table 2). However, XX runners presented a higher frequency of sudden injuries (*P* = 0.024), and the OR of suffering a muscle-type injury was 2.0 (0.51–7.79) times higher for XX than for RR runners and 3.52 (0.91–13.51) times higher for XX than for RX runners. Overall, XX runners were 2.86 (0.91–8.96) more prone to suffer muscle injuries that R-allele carriers (Fig 2). Considered only muscle injuries, the difference in the occurrence of injuries (10.0/6.0/18.2% for RR/RX/XX runners, *P* = 0.160) and in the rate of injuries (mean = 0.39/0.25/0.90, *P* = 0.071; median = 0.0/0.0/0.0 injuries per 1000 h of endurance running, *P* = 0.180) did not reach statistical significance.

## Discussion

In recent years, there has been considerable growth in the understanding of how genes and their expression are influencing the response to exercise training and the predisposition to sustain sports-related injuries [29]. Based on this rising knowledge, direct-to-the-consumer genetic testing is currently available on the market [30] aiming to optimize physical conditioning and to provide more effective injury prevention. The current genetic panels often include the characterization of the *ACTN3* R577X genotype, although the influence of α-actinin-3 deficiency on injury epidemiology has only been scarcely tested and with diverse outcomes [18]. In an attempt to gain further information on this issue, the present study investigated the influence of *ACTN3* genotypes on injury prevalence, incidence and conditions in marathoners during the year preceding to participating in a competitive marathon race. The primary outcomes of this investigation indicate that RR marathoners were 1.88 times more likely to suffer an injury related to endurance running training or competition when compared to X-allele carriers. In contrast, XX presented a higher frequency of sudden injuries and they were 2.86 more prone to sustain a muscle-type injury than R-allele carriers. Apart from these differences, the remaining injury-related variables were very similar in all three genotypes indicating that body location, severity, time of year, and possible cause of injury were unaffected by the *ACTN3* genotype.

Endurance running is one of the most popular physical activities in the world, but there exists a substantial injury prevalence with a range between 18.2 to 92.4% of injured runners, depending on the type and definition of the injury [1]. In the current investigation, 61 out of 139 endurance runners (44.2%) reported at least one injury during the year before the marathon race. Interestingly, the likelihood of suffering any injury related to endurance running was ~2 times higher in RR compared to any of the genotypes carrying the X allele (i.e., RX and XX). This finding is in agreement with the higher prevalence of sport-injuries previously reported in female athletes who were R-allele carriers vs. X-allele carriers [21]. Although the cause is not evident from our data, we can hypothesize that this finding may be due to the decreased range of motion in several joints and the increased passive hamstring stiffness found in RR individuals compared to XX counterparts [22]. In amateur marathoners, XX runners have higher muscle flexibility than RX runners and have higher ankle dorsiflexion values than RR runners [15]. Although it has been suggested that muscle flexibility neither improve nor decrease the likeliness of developing running-related injuries [31], extreme cases outside the normal range of flexibility might predispose to endurance running injuries. Taken together, this information might be indicative of a higher likelihood of suffering any type of endurance running injury in R-allele carriers, especially RR runners. Nevertheless, to determine whether this higher prevalence of endurance running injury in RR vs. XX runners is due to reduced muscle flexibility requires further confirmation.

Our analysis shows the opposite outcome if we only attend to muscle type injuries. In this case, the likelihood of sustaining a muscle injury was higher in XX endurance runners than in their RR and RX counterparts (Fig 1). The same findings were observed for sudden-onset injuries in elite soccer players [19, 20]. This outcome may suggest a lower capacity of α-actinin-3 deficient individuals (i.e., XX genotype) to resist muscle strain during sport-related activities. Individuals with the XX genotype, as opposed to those with the RX or RR genotypes, are unable to express α-actinin-3 in fast-type muscle fibers [32]. Although XX individuals compensate for the deficiency of α-actinin-3 with a higher expression of α-actinin-2 [9], this does not prevent a diminished muscle function in humans [33, 34]. In addition, α-actinin-3 deficient runners presented higher values of exercise muscle damage in endurance [17], and ultra-endurance events [35]. Animal investigations with *Actn3* knock-out mice have reported changes in the composition of the fast fiber Z-disc that alters their elastic properties, leading to increased susceptibility to contraction-induced muscle damage [36]. Thus, human and animal evidence suggests that the *ACTN3* XX genotype leads to reduced muscle performance that might end in a higher likelihood of muscle damage and injury, particularly in endurance runners. The design of specific programs to prevent muscle damage and injuries in α-actinin-3 deficient merits further investigation.

The current manuscript has some limitations that should be commented on. First, the current investigation only reported time loss injuries that required medical attention. This might have influenced the results of the investigation because it is likely that not all runners had the same access or sought medical care. Furthermore, this criterion excluded the recollection of data on injuries that, despite producing a time loss, were not diagnosed by a medical professional. Although the use of medical attention injuries might have biased the results to more severe injuries and in runners with high access to medical care, we selected this measure to increase the rigor of the data collection for each injury. A second limitation is that the frequency and rate of endurance running injuries found this investigation might have been influenced by selection bias. Runners more prone to injury might have been more willing to participate in the investigation, and the rates of these variables could have been overestimated. In any case, this limitation does not interfere with the *ACTN3* genotypes, and it is highly likely that the potential bias was equally distributed across genotypes.

In summary, α-actinin-3 deficiency, resulting from homozygosity in the null-X allele of the *ACTN3* gene, did not result in a higher prevalence or incidence of lower limb injury in endurance runners training for a marathon race. In fact, XX runners were two times less likely to suffer a non-contact endurance running injury than RR runners. However, the likelihood of suffering a muscle injury, especially with a sudden-onset, was twice as high in XX than in RR subjects. Thus, α-actinin-3 deficient runners might be more prone to sustain muscle type injuries in the lower limbs during the physical preparation for a marathon race. In this regard, it could be advisable that runners prone to suffering from running-related muscle injury engage in injury prevention programs to reduce their likelihood of suffering muscle injuries, while a higher incidence of this type of injury might be expected in runners with the XX genotype. Nevertheless, practitioners and end-users of sports-related genetic information should be aware of the current scarcity of large and scientifically sound studies related to this topic.
